# CCT and Cullin1 Regulate the TORC1 Pathway to Promote Dendritic Arborization in Health and Disease

**DOI:** 10.3390/cells13121029

**Published:** 2024-06-13

**Authors:** Erin N. Lottes, Feyza Ciger, Shatabdi Bhattacharjee, Emily A. Timmins, Benoit Tete, Tommy Tran, Jais Matta, Atit A. Patel, Daniel N. Cox

**Affiliations:** Neuroscience Institute, Georgia State University, Atlanta, GA 30303, USA

**Keywords:** dendrite development, chaperone, TORC1, E3 ubiquitin ligase, Huntingtin, *Drosophila*

## Abstract

The development of cell-type-specific dendritic arbors is integral to the proper functioning of neurons within their circuit networks. In this study, we examine the regulatory relationship between the cytosolic chaperonin CCT, key insulin pathway genes, and an E3 ubiquitin ligase (Cullin1) in dendritic development. CCT loss of function (LOF) results in dendritic hypotrophy in *Drosophila* Class IV (CIV) multi-dendritic larval sensory neurons, and CCT has recently been shown to fold components of the TOR (Target of Rapamycin) complex 1 (TORC1) in vitro. Through targeted genetic manipulations, we confirm that an LOF of CCT and the TORC1 pathway reduces dendritic complexity, while overexpression of key TORC1 pathway genes increases the dendritic complexity in CIV neurons. Furthermore, both CCT and TORC1 LOF significantly reduce microtubule (MT) stability. CCT has been previously implicated in regulating proteinopathic aggregation, thus, we examine CIV dendritic development in disease conditions as well. The expression of mutant Huntingtin leads to dendritic hypotrophy in a repeat-length-dependent manner, which can be rescued by Cullin1 LOF. Together, our data suggest that Cullin1 and CCT influence dendritic arborization through the regulation of TORC1 in both health and disease.

## 1. Introduction

It once was thought that the brain was isolated from the effects of starvation: at the start of the 20th century, Edward H. Dewey, M.D. asserted, “The brain is not only a self-feeding organ when necessary, but it is also a self-charging dynamo, regaining its exhausted energies entirely through rest and sleep” [1]. Cognitive symptoms were repeatedly connected to diabetes [2,3], but it was not until the discovery of neuronal insulin that the idea of the metabolically insulated brain was retired [4,5,6,7]. Research linking neurodegenerative disorders to insulin resistance has since highlighted the necessity of understanding how the insulin pathway modulates the brain in health and disease [8,9,10,11]. In this study, we examine the putative connections among three cytosolic mechanisms—chaperone activity, the TORC1 pathway, and ubiquitin ligase activity—which each coordinate dendritic arborization through the regulation of the cytoskeleton.

**Figure 1:**
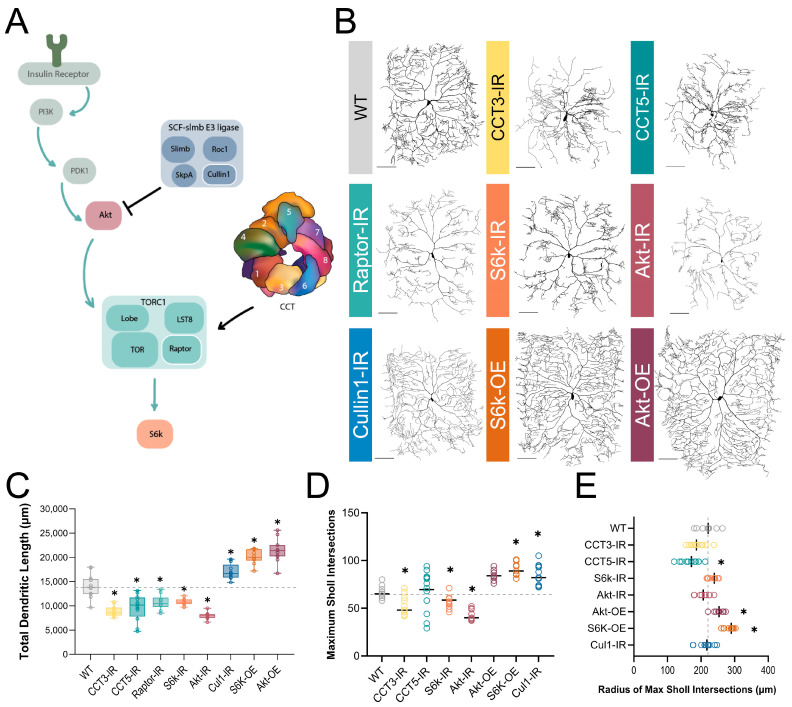
**CCT and the TORC1 pathway promote dendritic arborization**. (**A**) Schematic diagram of regulatory relationships between the insulin pathway, SCF complex, CCT, and TORC1 pathway, with the insulin pathway indicated by teal arrows. TORC1 is negatively regulated by Cullin1 and positively regulated by CCT. The upstream insulin pathway in green is displayed for context, but was not examined in this study. Individual components of the SCF and TORC1 complexes examined in this study are outlined in white. (**B**) Representative images of CIV neurons for key CCT and TORC1 pathway manipulations, with RNAi-mediated knockdown indicated with -IR and UAS-mediated overexpression with -OE. Scale bars = 100 µm (**C**) Total dendritic length of CCT and TORC1 pathway manipulations shown in comparison to a WT control. (**D**) Number of Sholl maximum intersections. (**E**) Radius (in µm) of Sholl maximum intersection for each genotype. Radii that have shifted a significant difference from control are indicated with an asterisk. In all panels * = *p* < 0.05, see Supplementary Table S2 for detailed statistics.

In *Drosophila melanogaster*, the chaperonin CCT (Complex Containing Tailless Complex Polypeptide 1 [TCP-1], also known as TRiC) is required for the dendritic development of Class IV (CIV) multi-dendritic (md) sensory neurons of the larval peripheral nervous system [12,13]. CCT is a chaperonin—an ATP-dependent chaperone—and has the canonical chaperonin “barrel” shape, composed of two repeating rings of eight subunits each: CCT1-8 (Figure 1A) [14,15]. Estimated to fold from 1 to 15% of cellular proteins, two of CCT’s most notable clients are cytoskeletal monomeric subunits actin and tubulin [16,17,18,19,20].

CCT physically interacts with proteins in the TORC1 pathway and folds Raptor, the regulatory component of TORC1 [21,22]. TORC1 is a part of the insulin pathway, operating downstream of Phosphatidylinositol-3 kinase (PI3K) and Akt kinase (Figure 1A), and has been long known to control cell size [23,24] and dendritic development in many organisms [24,25,26,27,28,29,30,31,32].

In this study, we investigate the regulatory roles of CCT, TORC1, and an E3 ubiquitin ligase in both conditions of normal development and proteinopathic disease states. Cullin1 is the scaffolding component of the Skp, Cullin, F-box (SCF) E3-ubiquitin ligase—previously shown to regulate TORC1 in dendritic pruning in *Drosophila* [33]. We establish, in vivo, that in normal development, dendritic arborization is mediated by a chaperonin (CCT) and E3 ubiquitin ligase component (Cullin1), both of which partially mediate dendritic complexity through the regulation of TORC1. TORC1 inhibition leads to dendritic hypotrophy, whereas TORC1 activation leads to dendritic hypertrophy.

CCT and TORC1 have also been examined as endogenous mediators of the cellular consequences of Huntington’s Disease (HD), a neurodegenerative disease caused by a polyglutamine expansion mutation. Targeted manipulations of CCT and TORC1 have been found to reduce aggregates and enhance cell viability in multiple model systems of HD [34,35,36,37]. TORC1 inhibition, via the application of rapamycin and similar drugs, has been shown to be neuroprotective in cell culture models of HD, as well as in both *Drosophila* and zebrafish photoreceptors with mutant Huntingtin [38,39,40,41]. However, the potential roles of TORC1 and CCT in regulating dendritic development in HD conditions have not been explored. In CIV neurons, we find that, although high repeat mutant Huntingtin (mHTT) expression results in dendritic hypotrophy and a loss of underlying microtubule signals, like that of TORC1 and CCT LOF, we do not find evidence that mHTT disrupts the TORC1–CCT dendritic arborization pathway in HD conditions. In contrast, Cullin1, when knocked down in mHTT conditions, increases dendritic complexity back to control levels, indicating it may play a separate role in HD-mediated dendritic hypotrophy.

## 2. Methods

### 2.1. Drosophila Husbandry and Stocks

The *Drosophila melanogaster* stocks used in this study were reared on a standard recipe of cornmeal, molasses, and agar media and maintained at 25 °C. Genetic crosses for live imaging and immunohistochemistry were reared at 29 °C. In all experiments, larvae were randomized for sex. The complete list of stocks and genetic lines used for this study is listed in Supplementary Table S1. In the main figures, all fly lines were crossed to *GAL4[477];ppk-GAL4::GFP*, except for in *mCherry:Jupiter* experiments, as described in the following methods, and select Supplemental Figures, as described in the legends.

### 2.2. Immunohistochemical Analysis

Larval dissection, mounting, and staining were performed as previously described [42,43]. Third instar larvae were stained for CCT, acetylated α-tubulin, and Raptor. Samples were fixed and then imaged with a Zeiss LSM780 Confocal microscope under 63× magnification using an oil immersion objective. Quantification of the fluorescent signal was performed by tracing CIV somas using the Zeiss Blue Lite Spline Contour tool and comparing Mean Intensity Values, which were normalized to area to control for the size of ROI. The primary antibodies used included: chicken anti-GFP (1:1000 dilution, Aves Labs, Davis, CA, USA), rabbit anti-phosphorylated S6k (1:300 dilution, Cell Signaling Technology, Danvers, MA, USA), rabbit anti-Raptor (1:200 dilution, Cell Signaling Technology, Danvers, MA, USA), mouse anti-acetylated α-tubulin (1:100 dilution, Santa Cruz Biotechnology, Dallas, TX, USA), mouse anti-Futsch (1:100 dilution, Developmental Studies Hybridoma Bank, Iowa City, IA, USA), rabbit anti-Huntingtin (1:200 dilution, Cell Signaling Technology, Danvers, MA, USA), mouse anti-CCT5 (1:200 dilution, GeneTex, Irvine, CA, USA), mouse anti-S6k (1:200 dilution, Proteintech, Rosemont, IL, USA), rabbit anti-phosphorylated Akt (1:200 dilution, Cell Signaling Technology, Danvers, MA, USA), rabbit anti-Cullin1 (1:200 dilution, Invitrogen), and mouse anti-β-tubulin IIA (1:500 dilution, Novus Biologicals, Centennial, CO, USA). The secondary antibodies used included: donkey anti-chicken 488 (1:2000 dilution, Jackson Immunoresearch, West Grove, PA, USA), donkey anti-mouse 555 (1:200 dilution, Life Technologies, Carlsbad, CA, USA), donkey anti-mouse 568 (1:200 dilution, Life Technologies, Carlsbad, CA, USA), donkey anti-mouse 647 (1:200 dilution, Life Technologies, Carlsbad, CA, USA), donkey anti-rabbit 568 (1:200 dilution, Life Technologies, Carlsbad, CA, USA), donkey anti-rabbit 647 (1:200 dilution, Life Technologies, Carlsbad, CA, USA), and donkey anti-rat Cy3 (1:200 dilution, Jackson Immunoresearch, West Grove, PA, USA).

### 2.3. Live Confocal Imaging, Neural Reconstructions, and Morphometric Analyses

Live imaging was performed using the Zeiss LSM780 Confocal as previously described on the third instar larvae [44,45]. Multiple gene-specific RNAi lines were examined for each genotype and validated using IHC and mutant analysis when possible. An MARCM analysis was performed as previously described [43,45]. To generate CIV neuron MARCM clones, *GAL^5-40^UAS-Venus:pm SOP-FLP#42;tubP-GAL80FRT40A* (2L MARCM) flies were crossed to *CCT4^KG09280^*,*FRT40A* mutant flies. Maximum-intensity projections of dendritic z-stacks were processed and the neurons were reconstructed, as previously described [46]. Quantitative morphological data (including the total dendritic length and Sholl analysis) were compiled using the Simple Neurite Tracer (SNT) plugin for FIJI [47,48]. Batch processing was completed using a custom FIJI macro and Rstudio script created by Dr. Atit A. Patel (Cox Lab), and the resulting data were exported to Excel (Microsoft, Version 16.0.1).

### 2.4. Live Multichannel Neural Reconstructions

Multichannel cytoskeletal reconstructions and related quantitative analyses were performed using the method described in [49] and implemented in [50] for CIV cytoskeletal analysis. All fly lines were crossed to *UAS-GMA::GFP;GAL4[477];UAS-mCherry::Jupiter* in these experiments. In brief, one primary branch and all connected distal branches in the same posterior quadrant were reconstructed for each neuron using Neutube [51], then, microtubule (MT) fluorescence was measured at distinct points along the dendritic arbor, averaged in 20 or 40 µm bins, and normalized to 1 for comparison to controls. The displayed *Jupiter::mCherry* fluorescence is shown as a ratio of the normalized fluorescence over the total path length within each bin and can be understood as the average normalized fluorescence along a single branch.

### 2.5. mHTT Aggregate Inclusion Body Analysis

Inclusion body aggregates of mHTT were manually quantified using the Zen Blue Lite software in neurons expressing *mHTTQ96-Cerulean* imaged live at 20× magnification. Neuron labels were coded for analysis to ensure blind conditions. The inclusion bodies were outlined using the Zen “Draw Spline Contour” tool and average areas compared across genotypes.

### 2.6. Experimental Design and Statistical Analyses

Statistical analyses were performed using GraphPad Prism 10. Error bars in the figures represent the standard error of the mean (SEM). All data were tested for normality using the Shapiro–Wilk normality test. The statistical tests performed included: unpaired *t*-test; one-way ANOVA with Dunnett’s, Šídák’s, or Tukey’s multiple comparison test (multiple comparison tests chosen based on Prism 10 recommendations); two-way ANOVA with Tukey’s multiple comparison test; Mann–Whitney *U* test; and Kruskal–Wallis test using Dunn’s multiple comparison test. Data points lying greater than two standard deviations above or below the mean were removed. A single asterisk is used in all graphs to denote significance (*p* ≤ 0.05), and detailed statistical results are available in Supplementary Table S2. 

## 3. Results

### 3.1. CCT LOF and Disruption of TORC1 Pathway Genes Results in Dendritic Hypotrophy

CCT is required for complex dendritic arbor formation in *Drosophila melanogaster* CIV md neurons [12,13], as we independently confirmed in this study via gene-specific RNAi and MARCM clonal analyses (Figure 1B–E and Figure S1A–E). LOFs of *CCT3* and *CCT5* both result in significant dendritic hypotrophy and will be used throughout this study to disrupt the CCT complex (Figure 1B–E). LOF of single CCT subunits has previously been found to reduce the expression of other CCT subunits [22,52,53], and we independently found via immunohistochemistry (IHC) that RNAi knockdown of *CCT4* (*CCT4-IR*) results in significant reductions in CCT5 expression (Figure S1F,G). Additionally, simultaneous RNAi knockdown of *CCT4* and *CCT5* results in significantly lower CCT5 expression than *CCT4-IR* or *CCT5-IR* alone (Figure S1F,G). Developmental time course analyses revealed that CIV dendritic hypotrophy with *CCT3-IR* or *CCT5-IR* first manifests at 72 h after egg laying (AEL)—as indicated by reductions in total dendritic length (TDL) that plateau later in larval development (Figure S1H).

**Figure 2:**
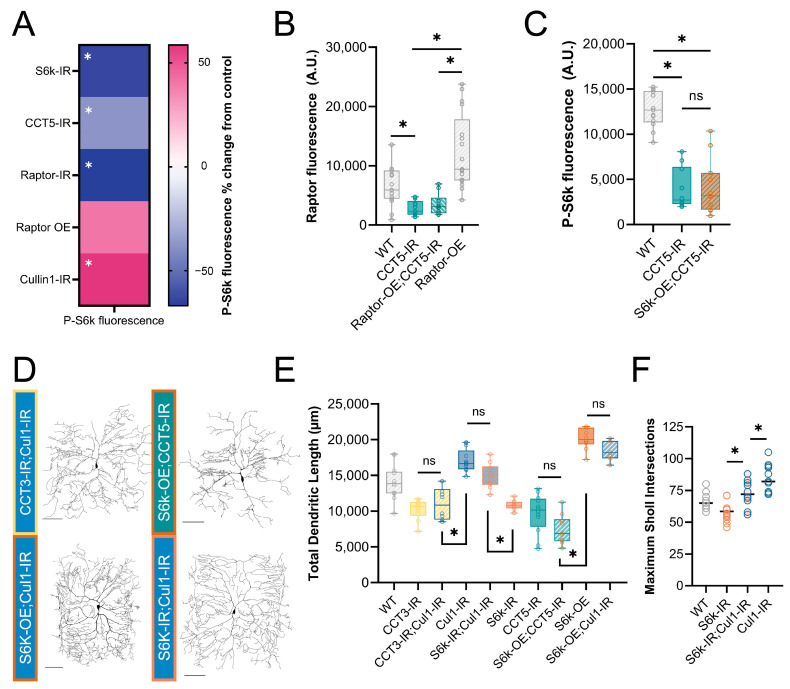
**CCT and Cullin1 regulate the TORC1 pathway in vivo**. (**A**) Heat map showing the percent change in P-S6k fluorescence for each genetic manipulation as compared to its proper control. See Figure S2I for representative images. (**B**) Raptor fluorescence is significantly decreased in *CCT5* LOF conditions and is not rescued by overexpression of Raptor. (**C**) P-S6k fluorescence levels are significantly decreased in *CCT5* LOF and are not rescued by overexpression of S6k. (**D**) Representative images of combined TORC1 genetic manipulations. Scale bars = 100 µm. (**E**) Total dendritic length in microns for WT and combined TORC1 genetic manipulations. (**F**) Number of Sholl maximum intersections for S6k and Cullin1 individual and combined LOF. In all panels * = *p* < 0.05, ns = not significant; see Supplementary Table S2 for detailed statistics.

Though many clients of CCT have been identified, only tubulin and actin have been examined alongside CCT in dendritic development [13]. There is evidence that CCT folds components of TORC1 in vitro and co-operates with the insulin pathway to regulate organ size in *Drosophila* [21,22]. Using RNAi, we knocked down key insulin pathway effector genes: *Akt*, *Raptor*, and *S6k*—an activator, a component, and a downstream effector of TORC1, respectively (Figure 1A). The efficacy of various RNAi knockdowns was confirmed by quantifying the fluorescence of each protein of interest in the wild-type (WT), control, and RNAi knockdown conditions. *Raptor-IR* resulted in significant reductions in Raptor fluorescence relative to the control (Figure S2A,B). *S6k-IR*, likewise, resulted in significant decreases in S6k (Figure S2C,D) and phosphorylated S6k (P-S6k) expression, the active form of S6k (Figure 2A and Figure S2I). *Akt-IR* also resulted in a significant decrease in phosphorylated Akt expression (Figure S2E,F). Akt, Raptor, and S6k LOF all resulted in significant dendritic hypotrophy, as measured by TDL (Figure 1B,C). Furthermore, the maximum number of Sholl intersections was significantly decreased in *Akt-IR* and *CCT3-IR* conditions (Figure 1D), indicative of decreased branch complexity; additionally, *CCT5-IR* led to a significant proximal shift in the Sholl maximum radius toward the soma (Figure 1E). Collectively, these data demonstrate that both CCT and the TORC1 pathway are required for CIV dendritic development.

### 3.2. TORC1 Hyperactivation Results in Dendritic Hypertrophy

There is evidence in multiple model organisms that TORC1 hyperactivation can result in increases in dendritic complexity [31,54,55]. In CIV neurons, the overexpression (OE) of key components of the TORC1 pathway result in an increased complexity of dendritic arbors (Figure 1B–E). *S6k OE* and *Akt OE* both result in significantly increased TDL, a significantly higher Sholl maximum, and a significantly, distally shifted Sholl maximum radius relative to controls (Figure 1B–E). In contrast, the overexpression of individual subunits of CCT does not produce any significant changes in TDL (Figure S1I), consistent with prior studies [12]. Additionally, the overexpression of single CCT subunits has been found to be insufficient to increase the levels of other CCT subunits in the complex [36,56].

Cullin1, a scaffolding component of the SCF complex, has previously been shown to regulate TORC1 activity in CIV dendrite pruning [33] through the inhibition of Akt (Figure 1A). Another component of SCF, SkpA, was also previously reported to produce dendritic hypertrophy under LOF conditions [12]. We find that, as in *SkpA-IR* and *Akt* OE, *Cullin1-IR* results in significantly increased TDL in CIV neurons (Figure 1B,C). The efficacy of *Cullin1* RNAi was confirmed via IHC, as *Cullin1* LOF leads to a significant reduction in Cullin1 fluorescence in CIV neurons (Figure S2G,H).

To validate the activation or inhibition of TORC1 through genetic manipulations of pathway components and cytosolic interactors, we stained for the downstream product of TORC1: phosphorylated S6k (P-S6k) (Figure 1A and Figure 2A). *Raptor-IR* and *CCT5-IR* significantly decrease P-S6k levels, confirming the disruption of TORC1 (Figure 2A). *Cullin1-IR* significantly increases the levels of P-S6k (Figure 2A), indicating that the knockdown of *Cullin1* disinhibits TORC1 to phosphorylate S6k (Figure 1A).

### 3.3. CCT Regulates Raptor Levels In Vivo

CCT was recently demonstrated to fold Raptor, the regulatory component of TORC1 [21]. We confirm that this regulatory relationship is conserved in *Drosophila melanogaster* larval sensory neurons. First, we find that *CCT5-IR* significantly decreases the levels of Raptor in CIV neurons from both WT and *Raptor* overexpression backgrounds (Figure 2B and Figure S2A,B). Though the overexpression of Raptor via *UAS-Raptor-HA* significantly increases Raptor fluorescence over WT levels (Figure S2A,B), it is insufficient to increase Raptor fluorescence levels significantly in a *CCT5-IR* background, as measured via IHC (Figure 2B).

To further confirm the requirement of CCT to sustain the TORC1 pathway, we overexpressed *S6k* in the *CCT5-IR* background (Figure 2C–E). We hypothesized that, if CCT was required for the TORC1-mediated phosphorylation of S6k, then S6k OE would not be sufficient to rescue either P-S6k levels or dendritic arborization. Indeed, S6k OE could not return the levels of P-S6k fluorescence to the WT conditions in a *CCT5-IR* background (Figure 2C). Likewise, the overexpression of S6k in *CCT5-IR* neurons was unable to rescue *CCT5*-LOF-induced dendritic hypotrophy (Figure 2D,E), revealing that CCT is necessary for *S6k*-OE-induced dendritic hypertrophy. These data indicate that CCT is required for Raptor expression and subsequent S6k phosphorylation through TORC1.

### 3.4. Cullin1 Regulates Dendritic Arborization through TORC1

*Cullin1-IR* significantly increases the levels of P-S6k fluorescence, as measured through IHC (Figure 2A). *Cullin1-IR* and *S6k* OE both lead to dendritic hypertrophy (Figure 1B–E). However, combining *Cullin1* knockdown and *S6k* overexpression in the same neurons does not further increase dendritic complexity, and there is no significant difference in TDL between the individual manipulations and the combined phenotype (Figure 2D,E).

We hypothesized that if *Cullin1* LOF were causing dendritic hypertrophy through the regulation of TORC1, then a loss of S6k function would rescue the *Cullin1-IR* hyper-arborization phenotype. Indeed, the combined *S6k-IR;Cullin1-IR* neurons display a TDL that does not significantly differ from WT (Figure 2E). *S6k-IR;Cullin1-IR* neurons show a significantly higher TDL than *S6k-IR* alone, but the TDL is not significantly different from *Cullin1-IR* alone (Figure 2D,E). As *Cullin1-IR* significantly increases the branching complexity from WT (Figure 1D) in addition to the TDL (Figure 1C), we postulated that *S6k-IR;Cullin1-IR* might display differences in numbers of branches without altering the overall TDL. Therefore, we examined the Sholl maximum intersections for each genotype, and found that the combined *S6k-IR;Cullin1-IR* has significantly more maximum intersections than *S6k-IR* and significantly fewer than *Cullin1-IR* (Figure 2F). Therefore, dendritic hypertrophy induced by *Cullin1* LOF requires WT S6k levels, supporting the hypothesis that *Cullin1* influences dendritic arborization through the regulation of the TORC1 pathway.

We further hypothesized that, if *Cullin1* LOF were causing dendritic hypertrophy through the regulation of TORC1, then it would be unable to recover any of the lost complexity in CCT LOF neurons, as we have established CCT is essential for TORC1 activity (Figure 2B,C). *Cullin1-IR* does not significantly change the TDL in a *CCT3-IR* background compared to *CCT3-IR* alone (Figure 2E), indicating that CCT function is required for the observed hypertrophy in *Cullin1* knockdown neurons.

### 3.5. TORC1 Pathway Disruption Results in Loss of Stable Microtubules

**Figure 3:**
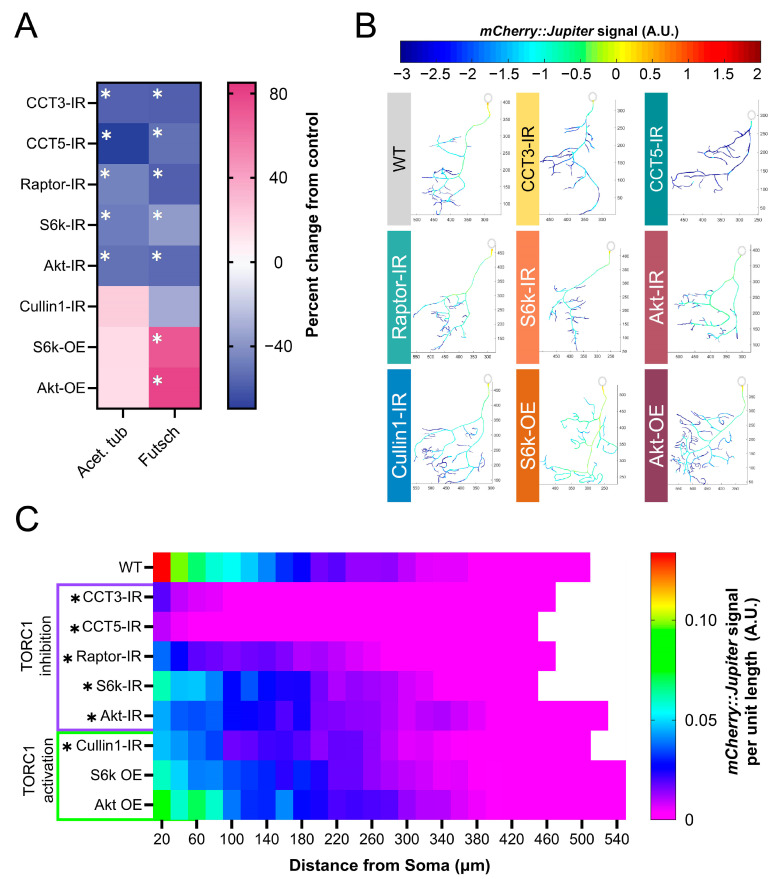
**TORC1 pathway manipulations alter underlying stable MT signal**. (**A**) Heat map showing percent change from control in acetylated α-tubulin and Futsch levels for each genetic manipulation. Each experimental condition was compared to WT control and appropriate statistical comparisons were performed (detailed in Supplementary Table S2). See Figure S3A,B for representative images. (**B**) Representative reconstructions of branches from WT and TORC1 genetic manipulations—normalized *mCherry::Jupiter* fluorescence is coded with the rainbow spectrum shown (A.U.). Scaled axes are provided in µm. (**C**) Heat map representing the average normalized, binned *mCherry::Jupiter* fluorescence along dendrites at increasing distances from the soma for each genotype. TORC1 inhibitions are marked in purple and TORC1 activations in green. Genotypes found to be significantly different along the dendritic arbor are marked with an asterisk. In all panels * = *p* < 0.05, see Supplementary Table S2 for detailed statistics.

CCT has been demonstrated to directly fold both α- and β-tubulin [20,57] and regulate stable microtubule (MT) levels in CIV dendrites [12,13]. We independently confirmed that CCT LOF leads to significant reductions in the underlying levels of stable MTs through several measures (Figure 3A–C and Figure S3A–D). Since inhibition of the TORC1 pathway causes significant dendritic hypotrophy, and TORC1 has been previously linked to cytoskeletal phenotypes [58], we predicted that there would be underlying cytoskeletal changes accompanying the loss of complexity.

We examined the levels of Futsch—a microtubule-associated protein (MAP)—acetylated α-tubulin, and βtubulin IIA in the soma of CIV neurons. Both Futsch and acetylated α-tubulin serve as markers of stable MTs [59,60,61,62], and β-tubulin IIA is an MT subunit known to be specifically folded by CCT [57].

In CIV neurons, the basal levels of acetylated α-tubulin are significantly reduced in *CCT3-IR*, *CCT5-IR*, *Raptor-IR*, *S6k-IR*, and *Akt-IR*, but are not significantly changed in *Cullin1-IR*, *S6k OE*, or *Akt OE* (Figure 3A and Figure S3A). Futsch is significantly decreased in TORC1 inhibition conditions: LOFs of *CCT3*, *CCT5*, *S6k*, *Raptor*, or *Akt* lead to significant reductions in Futsch fluorescence (Figure 3A and Figure S3B). In contrast, *Akt* and *S6k* OE lead to significant increases in the Futsch signal (Figure 3A and Figure S3B); however, *Cullin1-IR* does not show a significant change from the control.

*CCT3-IR* and *CCT5-IR* lead to significant decreases in β-tubulin IIA, as CCT LOF does for measures of MT stability (Figure 3A and Figure S3A–D). Although *Akt-IR* and *Raptor-IR* also significantly reduce β-tubulin IIA fluorescence, surprisingly, *S6k-IR* significantly increases the overall levels of β-tubulin IIA compared to WT neurons. Interestingly, *Cullin1-IR* also significantly decreases β-tubulin IIA fluorescence (Figure S3C,D).

We further confirm that TORC1 LOF reduces stable MT levels throughout the dendritic arbor through the use of a fluorescent reporter coupled to the MT-associated protein Jupiter (*UAS-Jupiter::mCherry*) [12]. CCT LOF (*1*-, *3*-, and *5-IR*) results in the steepest decline in *Jupiter::mCherry* signal (Figure 3B,C). Similar to the effects on acetylated α-tubulin and Futsch, losses of *Raptor*, *S6k*, and *Akt* also significantly decrease the *Jupiter::mCherry* signal; however, *Akt* OE and *S6k* OE do not significantly alter the *Jupiter::mCherry* signal. Interestingly, *Cullin1-IR* significantly decreases the *Jupiter::mCherry* signal, despite also resulting in hyper-arborization (Figure 3B,C). In general, we find that TORC1 inhibition significantly decreases the MT signal along the dendritic arbor, and that TORC1 hyperactivation significantly increases select markers of the MT signal (e.g., Futsch), while most are not significantly changed from WT.

### 3.6. Mutant Huntingtin Expression Leads to Repeat-Length-Dependent Reduction in Branch Complexity and Underlying Microtubule Loss

**Figure 4:**
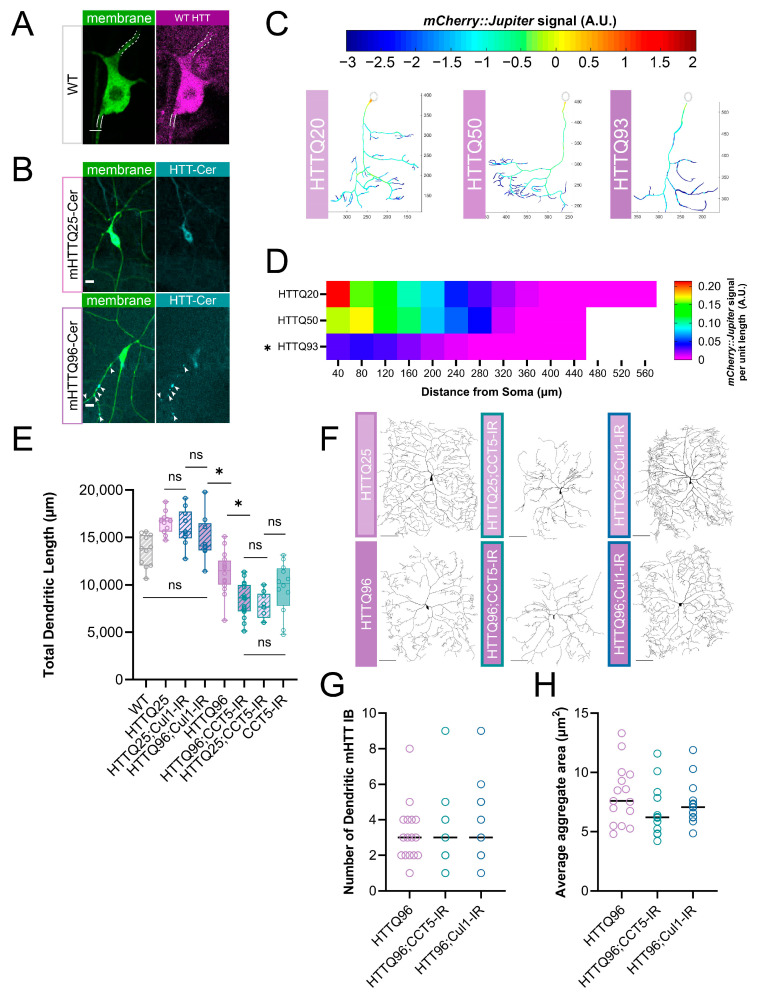
**Expression of mHTT leads to dendritic hypotrophy parallel to TORC1 pathway**. (**A**) Representative image of WT HTT staining in CIV neuron—dendrite marked by dashed white lines, axon by solid lines. Scale bar = 3 µm. (**B**) Representative images of mHTT25-Cerulean and mHTT96-Cerulean shown with aggregate inclusion bodies marked by white arrows for mHTT96-Cerulean. Scale bar = 10 µm. (**C**) Representative reconstructions of branches from WT and TORC1 genetic manipulations—normalized *mCherry::Jupiter* fluorescence is coded with the rainbow spectrum shown (A.U.) (**D**) Heat map representing the average normalized, binned *mCherry::Jupiter* fluorescence along dendrites at increasing distances from the soma for overexpressions of mHTT 20, 50, and 93 repeats. Genotypes found to be significantly different along the dendritic arbor are marked with an asterisk. (**E**) TDL of *Cullin1-IR* and *CCT5-IR* in both *mHTTQ25* and *mHTTQ96* backgrounds displayed as percent change from WT control. *CCT5-IR* decreases both *mHTTQ96* and *mHTTQ25* neurons to far lower than WT, while *Cullin1-IR* rescues mHTT96 hypotrophy to WT levels. CCT5-IR alone is not significantly different from either *mHTTQ25;CCT5-IR* or *mHTTQ96;CCT5-IR.* All genotypes vary significantly from WT except for *mHTTQ96;Cullin1-IR.* (**F**) Representative images of CIV dendritic morphology in combined *HTT* and *CCT5-IR* or *Cullin1-IR* combinations. Scale bars = 100 µm. (**G**) Number of mHTT aggregate IBs does not change due to *CCT5* or *Cullin1* LOF. (**H**) mHTT aggregates in mHTTQ96Cerulean conditions do not change in average area due to *CCT5* or *Cullin1* LOF. In all panels * = *p* < 0.05, ns = not significant; see Supplementary Table S2 for detailed statistics.

Though CCT and TORC1 clearly regulate dendritic arborization during normal development, there is also great interest in examining the putative relationships of these complexes with proteinopathic disease. Several studies have connected CCT to the regulation of mutant Huntingtin (mHTT) protein, and there is some evidence that a loss of wild-type HTT disrupts neuron formation [63,64]. *Drosophila melanogaster* has been used to model many HD-related phenomena, such as motor deficits, circadian rhythm changes, metabolic precursors of disease, mHTT aggregate spreading in the brain, and more [65,66,67,68,69]. Using UAS-mediated constructs of mutant Huntingtin (see Supplementary Table S1), we expressed human mHTT in CIV neurons and quantified the gross morphology of the resultant dendritic arbors. Shorter repeats of mHTTQ20 and mHTTQ50 do not significantly alter the arbor complexity, however, the expression of mHTTQ93 and mHTTQ120 significantly reduces the TDL from WT (Figure S4A,B). We also examined the HTT distribution in CIV neurons and identified somatic and dendritic expressions (Figure 4A). Consistent with WT HTT expression, the induced expression of mHTT96Cer shows clear expressions in soma and dendrites (Figure 4B). Though mHTT misexpression lines have a significantly higher overall HTT expression than WT (Figure S4G,H), neurons expressing *UAS-mHTTQ25-Cerulean* do not display apparent puncta (Figure 4B). In contrast, the expression of *UAS-mHTTQ96-Cerulean* results in aggregate inclusion bodies (IBs) of mHTT forming in the dendritic arbor (Figure 4B).

WT HTT is thought to be involved in cellular trafficking, and there is evidence that mHTT expression can destabilize MTs [69,70]. We predicted that there would be underlying cytoskeletal deficits in these neurons similar to the phenotypes we observe in *CCT* and *TORC1* LOF neurons. The expression of *mHTTQ96* significantly decreases the Futsch fluorescence levels in the soma, as measured via IHC (Figure S4C,D). Live imaging of *Jupiter::mCherry* reveals that the expression of *mHTTQ50* does not significantly reduce stable MT signals, but the expression of *mHTTQ93* significantly reduces stable MT signals across the arbor (Figure 4C,D) as compared to the non-phenotypic mHTTQ20. Overall, the expression of high repeats of mHTT results in dendritic hypotrophy and underlying losses of stable MTs in CIV neurons.

### 3.7. Cullin1 LOF Rescues mHTT-Mediated Gross Morphological Phenotype, While CCT LOF Shows No Additive Effect

A large number of previous studies have implicated CCT in the direct regulation of mHTT [34,36,37,56,71,72]; therefore, we first sought to answer whether CCT regulates wild-type Huntingtin. We found that *CCT5-IR* leads to a significant decrease in the soma levels of wild-type *Drosophila* Huntingtin, as measured through IHC (Figure S4E,F).

Previous in vitro evidence suggests that CCT may work to clear mHTT aggregates [34,37,56,71,73,74], thus, we predicted that CCT LOF may lead to a higher aggregate load in dendrites in vivo. However, CCT LOF in a *UAS-mHTTQ96-Cerulean* background does not lead to a significant change in IB number or size (Figure 4G,H). Additionally, *CCT5-IR* in an *mHTT96* background does not potentiate the loss of the Futsch signal, which is already significantly reduced in mHTTQ96 neurons (Figure S4C,D). Though CCT is required for WT HTT expression and has been found to physically interact with mHTT aggregates in vitro, at our current resolution of analyses, CCT LOF does not appear to exacerbate IB appearance.

TORC1 activity has also been explored as an avenue for mHTT clearance, mainly through inhibition via the application of rapamycin [35,75,76]. Given HTT’s many connections to both CCT and TORC1, we predicted that the expression of mHTT may influence dendritic arborization through the disruption of the insulin pathway and cytosolic interactors CCT and Cullin1. Interestingly, the knockdown of *Cullin1* in an *mHTTQ96* background increases the TDL significantly from *mHTT96* alone, returning it to WT levels (Figure 4E,F). Despite the rescuing of dendritic hypotrophy, when *Cullin1* is knocked down in a *UAS-mHTTQ96-Cerulean* background, neither the median number nor aggregate size of mHTT IBs change (Figure 4G,H). Overall, though we found that CCT5 is required for WT HTT levels, and that CCT1 and HTT, as well as mHTT, are both expressed in the cytoplasm of CIV soma and dendrites, we did not find that CCT LOF and mHTT expression resulted in an additive phenotype. Further, *CCT5-IR* expressed alone does not show a significantly different TDL from either *mHTTQ25;CCT5-IR* or *mHTTQ96;CCT5-IR* (Figure 4E,F). Interestingly, we did find that *Cullin1-IR* was sufficient to rescue mHTTQ96-mediated defects in TDL, though it did not significantly affect the appearance of mHTT dendritic IBs.

## 4. Discussion

### 4.1. A TORC1 Cytosolic Network Regulates Dendritic Development and the Underlying MT Cytoskeleton

The TORC1 pathway has many cytosolic interactors; we illuminated the roles of two, CCT and Cullin1, in dendritic development. Previous work established that CCT subunits are required for CIV dendritic arbor formation [12,13], and we confirmed that CCT LOF results in dendritic hypotrophy with underlying stable MT deficits. Though CCT directly folds actin and tubulin monomers, we predicted that its contribution to dendritic arborization may also extend to secondary regulators of the dendritic arbor, such as TORC1. TORC1 has been found to regulate dendritic arbors in mammalian dopaminergic neurons [31,32,54], and was recently found to be regulated by CCT in both *Drosophila* and human cell cultures [21,22,77]. We confirmed, in vivo, that CCT is required for WT levels of Raptor and P-S6k in *Drosophila* CIV neurons. Raptor overexpression was not sufficient to rescue the decreased levels of Raptor in a *CCT5-IR* background (Figure 2B). Similarly, S6k overexpression failed to increase the P-S6k levels in a *CCT5-IR* background (Figure 2C). Together, these data support previous findings that CCT expression is necessary for TORC1 assembly and function.

Cullin1, which was previously demonstrated to regulate TORC1 in dendritic pruning [33], inhibits TORC1 activity in CIV neurons. *Cullin1-IR* significantly increases the P-S6k levels (Figure 2A) and dendritic complexity, mirroring the phenotypes of TORC1 pathway overexpression. When S6k is knocked down in Cullin1 LOF neurons, dendritic complexity is returned to WT levels (Figure 2E,F). These results indicate that Cullin1 works to inhibit dendritic complexity via the regulation of TORC1 in normal developmental conditions.

TORC1 LOF results in dendritic hypotrophy, while TORC1 hyperactivation results in dendritic hypertrophy (Figure 1B–E). The hypotrophy resulting from both LOF of TORC1 and CCT is mirrored by underlying losses of stable MT markers in the soma, such as Futsch and acetylated α-tubulin, as well as the *Jupiter::mCherry* signal throughout the dendritic arbor (Figure 3A–C). A notable difference is that CCT, Raptor, and Akt LOF significantly reduce the β-tubulin IIA signal in the soma, while S6k LOF significantly increases the β-tubulin IIA signal (Figure S3C). The β-tubulin IIA antibody used in our experiments is not specific to either free or incorporated β-tubulin IIA, so the production of free β-tubulin IIA could create increased fluorescence, even in a cell with reduced stable MTs.

Our gross morphological findings of TORC1 LOF coincide tightly with those of a recent study demonstrating that changes in nutrition result in CIV dendritic hyper-arborization and subsequent changes in cell sensitivity and larval behavior [55]. In this study, Akt, TOR, and S6k were all found to be required for hyper-arborization induced by a low-yeast diet. The levels of phosphorylated Akt were increased in low-yeast diet conditions, and the overexpression of Akt increased dendritic complexity. *Akt* LOF and OE have been established to decrease and increase CIV dendritic coverage, respectively [78]. In our work, LOFs of Akt and S6k produced dendritic hypotrophy (Figure 1B–E), and while we found that there are underlying MT deficits in TORC1 LOF conditions (Figure 3A–C), it remains to be seen if the cytoskeletal phenotypes are inducible through diet changes.

Manipulations inducing TORC1 hyperactivity or the disinhibition of TORC1 all result in hypertrophy, but have variable effects on MT markers. TORC1 has been previously found to regulate the Cytoplasmic Linker Protein of 170 kDA (CLIP-170), which interacts with IQ motif-containing GTPase-activating protein 1 (IQGAP1) to coordinate the crosslinking of MT and actin [58], thereby supporting dendritic growth. Additionally, S6k has been found to be necessary for the stress-evoked acetylation of α-tubulin in mouse embryonic fibroblasts [79], but the mechanism connecting S6k to tubulin acetylation is still unclear. We found that TORC1 hyperactivation through *S6k* OE and *Akt* OE increases Futsch levels; however, the *Cullin1-IR*-mediated disinhibition of TORC1 does not show the same MT phenotypes, despite displaying a similar dendritic hypertrophy (Figure 3A–C). Although *Cullin1-IR* results in no significant changes in either acetylated α-tubulin or Futsch levels, it does lead to a significant decrease in the Jupiter signal. It may be that the loss of *Cullin1* reduces the expression or attachment of MAPs to MTs without affecting the underlying MT stability. Though we provide evidence that CCT, Cullin1, and TORC1 regulate stable MT expression, much more work will be needed to understand the mechanisms by which this occurs.

### 4.2. TORC1 Cytosolic Network and mHTT Interact in the Regulation of Dendritic Arbors

TORC1 has also been extensively investigated with respect to proteinopathies, including Huntington’s Disease (for recent review see [80]), as has CCT [36,37,53,56,71,72,74]. Similar to the TORC1 and CCT LOF phenotypes, the expression of high repeat human mHTT in CIV neurons reduces dendritic complexity and the underlying stable MT signal (Figure 4C,D and Figure S4A–D). We observed both WT and mutant HTT in multiple neuronal compartments; furthermore, the expression of mHTTQ96-Cerulean and mHTTQ120-HA produced large aggregate IBs in dendritic arbors, similar to those previously reported in [65] (Figure 4A,B).

We also carried out genetic interaction studies between CCT and mHTT expression. When combined, *CCT5-IR* and *mHTTQ96* expression do not display an additive dendritic phenotype: arbor complexity in an mHTT96 background is reduced to the same level as that in neurons with both mHTT25 and *CCT5-IR* (Figure 4E,F). Furthermore, *CCT5-IR* did not induce a change in the size or number of mHTT dendritic puncta in the *mHTTQ96-Cerulean* background (Figure 4G,H). This is in contrast with previously published iPSC data, which found that the LOF of individual CCT subunits triggered the aggregation of mHTT [36]. CIV neurons form IBs upon the expression of high repeat mHTT, whereas iPSCs expressing mHTT require heat or proteostatic stress to induce IB formation, which may explain this discrepancy. There may also be changes in the IBs that are not evident at our current resolution, such as the organization of mHTT within the IB or changes in the temporal dynamics of IB formation.

Cullin1, a cytosolic inhibitor of TORC1, does show genetic interaction with mHTT expression: when we knocked down *Cullin1* in *mHTTQ96* neurons, there was a significant increase in dendritic complexity, restoring the TDL to WT levels (Figure 4E,F). Unexpectedly, in this same genetic background, we did not observe any changes in the dendritic IB size or number (Figure 4G,H). We predicted that *Cullin1-IR* would lead to an increase in IB number or size for two reasons. First, Cullin1, as part of an E3 ubiquitin ligase, helps to ubiquitinate proteins for degradation [81], and *Cullin1* LOF has been linked to an increased protein aggregate load [82,83]. Second, *Cullin1-IR* leads to TORC1 disinhibition, which reduces autophagic activity [84]. Previous studies have shown that rapamycin application—the inhibition of TORC1—reduces mHTT aggregation in *Drosophila* ommatidia and mammalian cells [38,39], and other studies have shown that the inhibition of mTORC1 ameliorates mHTT pathology through increased autophagic activity [39,67,80,85]. There is evidence that the SCF complex is down-regulated in Parkinson’s Disease, Huntington’s Disease, and Spinal-Cerebellar Ataxia Type 3, and that further *Cullin1* LOF exacerbates aggregate phenotypes [82,83,86,87]. However, we found that, in CIV neurons, *Cullin1* knockdown increases dendritic complexity while the dendritic IB load remains unchanged. Therefore, it is possible that the role of Cullin1 in the suppression of complex dendritic development and its role in promoting the degradation of protein aggregates are carried out through distinct cellular pathways. There are undoubtedly several mechanisms by which complexes like TORC1, CCT, and SCF could interact with protein aggregates, providing fertile ground for future studies.

In summary, our study shows that CCT regulates TORC1 in vivo to promote dendritic arborization in homeostatic development. We further demonstrate that Cullin1 inhibits TORC1 in vivo to suppress dendritic arborization. At the cytoskeletal level, TORC1 hypoactivation leads to underlying stable MT deficits, while hyperactivation and disinhibition through *Cullin1* knockdown have distinct MT phenotypes. In proteinopathic disease conditions, high repeats of mHTT lead to dendritic hypotrophy, and *Cullin1* LOF can rescue mHTT-induced hypotrophy, though neither *Cullin1* LOF nor *CCT* LOF significantly alter mHTT aggregate IB expression. Our data, together with the previous literature, demonstrate the conserved roles of TORC1, CCT, and Cullin1 in dendritic regulation in healthy and diseased neurons.

## Data Availability

The original contributions presented in the study are included in the article/Supplementary Materials, further inquiries can be directed to the corresponding author.

## Supplementary Materials

The following supporting information can be downloaded at: https://www.mdpi.com/article/10.3390/cells13121029/s1, Figure S1: CCT subunit LOF results in significant dendritic hypotrophy and underlying loss of stable MTs; Figure S2: Evidence of RNAi efficacy and representative images of TORC1 pathway regulation; Figure S3: Representative images related to Figure 3 and effects of TORC1 pathway on β-tubulin IIA; Figure S4: mHTT aggregates are not affected by co-expression of CCT5-IR; Table S1: Complete list of stocks and genetic lines used for this study; Table S2: Statistical analyses performed for all figures.
