# Supplementary material for: CCT and Cullin1 Regulate the TORC1 Pathway to Promote Dendritic Arborization in Health and Disease
Source: Cells. 2024 Jun 13;13(12):1029. doi: 10.3390/cells13121029 (PMC11201622; doi:10.3390/cells13121029)
Supplement: Supplementary file 1 [file cells-13-01029-s001.zip › Supplementary_Figure_Legends.pdf]

Supplementary Figure Legends

Figure S1. CCT subunit LOF results in dendritic hypotrophy and underlying loss of stable MTs. (**A**) Loss of individual CCT subunits results in significant decreases in TDL from WT controls. *CCT4^{KG09280}* and the 40A empty FRT control were crossed to MARCM 40A FLP (*GAL$^{5-40}$UAS-Venus:pm SOP-FLP#42;tubP-GAL80FRT40A* [2L MARCM] DRGC 109947) as described in the Methods. (**B**) Homozygous CCT4 MARCM mutant clones show significantly decreased TDL from control. (**C**) Representative images of CCT4 homozygous MARCM mutant CIV clones vs. control CIV MARCM clones (40A empty FRT) Scale bar = 100 μm. (**D**) Number of Sholl maximum intersections. (**E**) Radius (in μm) of Sholl maximum intersection for each genotype. Radii that have shifted a significant difference from control are indicated with an asterisk. (**F**) RNAi of *CCT4* or *CCT5* lead to a significant reduction in CCT5 fluorescence relative to WT as obtained through IHC. Combined knockdown of both *CCT4* and *CCT5* significantly reduces CCT5 expression from either knockdown alone. (**G**) Representative images of the preceding graph. Scale bar = 5 μm. (**H**) TDL of neurons at 24, 48, 72, and 96 hours after egg lay (AEL) reveal significant decreases from WT in both *CCT5-IR* and *CCT3-IR* starting at 72 hours AEL. In this experiment *UAS-CCT3-IR;ppk-GAL4* and *GAL4^{477};UAS-CCT5-IR* were crossed to *nanos-GAL4;ppk-hCD4-tdTOMATO* and compared to *+;ppk-GAL4* and *GAL4^{477};+* crossed to *nanos-GAL4;ppk-hCD4-tdTOMATO* as controls, respectively. (**I**) Overexpression of individual CCT subunits (CCT2, CCT4, or CCT5) does not significantly alter TDL from their relevant control. CCT2-OE and drosCCT4 were crossed to *GAL4^{477};ppk-GAL4::GFP* and compared to *ORR* crossed to *GAL4^{477};ppk-GAL4::GFP* as control. CRISPR-mediated overexpression line *UAS-CCT5-TOE* was crossed to *dcas9;ppk-GAL4::GFP* and compared to *ORR* crossed to *dcas9;ppk-GAL4::GFP* as control. In all panels * = $p < 0.05$, see **Supplementary Table S2** for detailed statistics.

Figure S2. Evidence for RNAi efficacy and representative images of TORC1 pathway regulation. (**A**) Heat map showing percent change in Raptor fluorescence of CCT5-IR or Raptor-IR knockdowns, as well as Raptor OE as compared to controls. (**B**) Representative images of genetic manipulations and appropriate controls for the preceding heat map. (**C**) S6k fluorescence is significantly reduced in *S6k-IR* conditions as compared to WT. (**D**) Representative images for WT and *S6k-IR*. (**E**) P-Akt fluorescence is significantly reduced in *Akt-IR* conditions. (**F**) Representative images for WT and *Akt-IR*. (**G**) Cullin1 fluorescence is significantly reduced in *Cullin1-IR* conditions as compared to WT. (**H**) Representative images for WT and *Cul1-IR*. (**I**) Representative images of genetic manipulations and appropriate controls for the heat map in Fig 2A. (**J**) Representative images of genetic manipulations and control for Fig 2B. (**K**) Representative images of genetic manipulations and control for Fig 2C. All scale bars = 5 μm. In all panels * = $p < 0.05$, see **Supplementary Table S2** for detailed statistics.

Figure S3. Representative images for Fig 3 and effects of TORC1 pathway on β -tubulin IIA. (**A**) Representative images of genetic manipulations and appropriate controls for the acetylated α-tubulin heat map in Fig 3A. (**B**) Representative images of genetic manipulations and appropriate controls for the Futsch heat map in Fig 3A. (**C**) Heat map showing percent change in β -tubulin IIA for each genetic manipulation. Each experimental condition was compared to WT control and

appropriate statistical comparisons were performed. (**D**) Representative images of genetic manipulations and appropriate controls for the β -tubulin IIA heat map in Fig S3D. Scale bars = 5 μm. In all panels * = $p < 0.05$, see **Supplementary Table S2** for detailed statistics.

Figure S4. mHTT aggregates are not affected by co-expression of CCT5-IR. (**A**) Representative images of CIV neurons expressing mHTT polyQ repeat transgenes reveal repeat-length dependent dendritic hypotrophy. Scale bars = 100 μm. (**B**) TDL is significantly reduced from control in neurons expressing mHTT93 or mHTT120 CAG repeats. (**C**) Representative images of Futsch levels in WT, HTTQ96, and CCT5-IR neurons. Scale bar = 5 μm. (**D**) Fluorescent levels of Futsch are significantly reduced in mHTTQ96 conditions and are not significantly changed by additional *CCT5-IR* expression. (**E**) Representative images of HTT levels in WT and CCT5-IR neurons. Scale bar = 5 μm. (**F**) WT HTT fluorescence is significantly reduced in *CCT5* LOF conditions. (**G**) Expression of mHTT25-Cerulean or mHTT96-Cerulean both result in significant increases in HTT fluorescence from WT. (**H**) Representative images of HTT levels in WT, HTTQ25, and HTTQ96. Scale bar = 5 μm. In all panels * = $p < 0.05$, see **Supplementary Table S2** for detailed statistics.
