# Supplementary material for: CCT and Cullin1 Regulate the TORC1 Pathway to Promote Dendritic Arborization in Health and Disease
Source: Cells. 2024 Jun 13;13(12):1029. doi: 10.3390/cells13121029 (PMC11201622; doi:10.3390/cells13121029)
Supplement: Supplementary file 1 [file cells-13-01029-s001.zip › Supplementary_Table_S1.pdf]

**Shorthand**	**Full Genotype**
WT	*Oregon R (ORR) (B5)*
CCT3-IR	*UAS-CCT3-IR (v106093)*
CCT5-IR	*UAS-CCT5-IR (B41818)*
Raptor-IR	*UAS-Raptor-IR (B34814)*
S6k-IR	*UAS-S6k-IR (B57016) and (B41702)*
Akt-IR	*UAS-Akt-IR (B8191)*
Cullin1-IR	*UAS-Cullin1-IR (B36601)*
S6k-OE	*UAS-S6k (B6910)*
Akt-OE	*UAS-Akt1 (B8191)*
Raptor OE	*UAS-Raptor-HA (B53726)*
Raptor-OE;CCT5-IR	*UAS-Raptor-HA;UAS-CCT5-IR*
S6k-OE;CCT5-IR	*UAS-S6k;UAS-CCT5-IR*
S6k-OE;Cul1-IR	*UAS-S6k;UAS-Cullin1-IR*
S6k-IR;Cul1-IR	*UAS-S6k-IR;UAS-Cullin1-IR*
CCT3-IR;Cul1-IR	*UAS-CCT3-IR;UAS-Cullin1-IR*
HTTQ20	*UAS-human HTTQ20 (B68412)*
HTTQ50	*UAS-human HTTQ50 (B68413)*
HTTQ93	*UAS-human HTTQ93 (B68418)*
HTTQ25/HTTQ25Cer	*UAS-human HTTQ25-Cerulean (B58360)*
HTTQ96/HTTQ96Cer	*UAS-human HTTQ96-Cerulean (B56771)*
HTTQ25;Cul1-IR	*UAS-human HTTQ25-Cerulean;UAS-Cullin1-IR*
HTTQ96;CCT5-IR	*UAS-human HTTQ96-Cerulean;UAS-CCT5-IR*
HTTQ25;CCT5-IR	*UAS-human HTTQ25-Cerulean; UAS-CCT5-IR*
CCT1-IR	*UAS-CCT1-IR (B32854)*
CCT2-IR	*UAS-CCT2-IR (B34711)*
CCT4-IR	*UAS-CCT4-IR (v22154)*
CCT6-IR	*UAS-CCT6-IR (B43146)*
CCT7-IR	*UAS-CCT7-IR (B34931)*
CCT8-IR	*UAS-CCT8-IR (v103905)*
40A empty FRT	*P{Car20y}25F;P{neoFRT}40A (B1816)*
$CCT4^{KG09280}$	*ey-FLP1* FRT^{40A} $CG5525^{KG09280}$*, (DGRC 111690)*
CCT4-IR;CCT5-IR	*UAS-CCT4-IR (v106099);UAS-CCT5-IR*
CCT2-OE	*UAS-CCT2-EGFP (B53755)*
*drosCCT4	*UAS-Drosophila CCT4 (k10379)*
CCT5 TOE	*CCT5 (guide RNA) snRNA;U6:96Aa, snRNA:U6:96Ac (B78122)*
**Source Abbreviations**	**Full Center Name**
B	Bloomington Drosophila Stock Center
v	Vienna Drosophila Resource Center
DRGC	Kyoto Drosophila Stock Center

**Gift of Dr. Kwang-Wook Choi, KAIST, South Korea*

Fly lines were crossed to *GAL4^{477};ppk-GAL4::GFP*, with the following exceptions:

In *mCherry:Jupiter* experiments (Figs 3B-C, 4C-D) lines were crossed to *UAS-GMA::GFP;GAL4^{477};UAS-mCherry::Jupiter.*

For MARCM analysis (Fig S1B-C), CCT4^{KG09280} and the 40A empty FRT control were crossed to MARCM 40A FLP (*GAL$^{5-40}$UAS-Venus:pm SOP-FLP#42;tubP-GAL80FRT40A [2L MARCM] DRGC 109947)* as described in the Methods.

For developmental morphological analysis (Fig S1H), *UAS-CCT3-IR;ppk-GAL4* and *GAL4^{477};UAS-CCT5-IR* were crossed to *nanos-GAL4;ppk-hCD4-tdTOMATO* and compared to *+;ppk-GAL4* and *GAL4^{477};+* crossed to *nanos-GAL4;ppk-hCD4-tdTOMATO* as controls, respectively.

CRISPR-mediated overexpression line *UAS-CCT5-TOE* was crossed to *dcas9;ppk-GAL4::GFP* and compared to *ORR* crossed to *dcas9;ppk-GAL4::GFP* as control (Fig S1I).
