# Supplementary material for: CCT and Cullin1 Regulate the TORC1 Pathway to Promote Dendritic Arborization in Health and Disease
Source: Cells. 2024 Jun 13;13(12):1029. doi: 10.3390/cells13121029 (PMC11201622; doi:10.3390/cells13121029)
Supplement: Supplementary file 1 [file cells-13-01029-s001.zip › Supplementary_Table_S2.pdf]

Comparison	Passed Shapiro-Wilk Normality Test	Statistical test used	Sig	p-value	Number of neurons (N)
**Fig 1C (TDL)**		One-way ANOVA & Dunnett's			
WT vs. CCT3-IR	Yes		****	<0.0001	13, 11
WT vs. CCT5-IR	Yes		****	<0.0001	13, 12
WT vs. Raptor-IR	Yes		***	0.0002	13, 10
WT vs. S6k-IR	Yes		****	<0.0001	13, 13
WT vs. Akt-IR	Yes		****	<0.0001	13, 10
WT vs. Cul1-IR	Yes		***	0.0003	13, 13
WT vs. S6k-OE	Yes		****	<0.0001	13, 10
WT vs. Akt-OE	Yes		****	<0.0001	13, 11
**Fig ID (Sholl Maximum)**		One-way ANOVA & Dunnett's			
WT vs. CCT3-IR	Yes		*	0.0219	9, 11
WT vs. CCT5-IR	Yes		ns	>0.9999	9, 14
WT vs. S6k-IR	Yes		ns	0.2517	9, 14
WT vs. Akt-IR	Yes		****	<0.0001	9, 10
WT vs. Akt-OE	Yes		**	0.0012	9, 11
WT vs. S6k-OE	Yes		****	<0.0001	9,10
WT vs. Cul1-IR	Yes		**	0.0027	9,12
**Fig 1E (Sholl Radius)**		One-way ANOVA & Dunnett's			
WT vs. CCT3-IR	Yes		ns	0.0522	9, 11
WT vs. CCT5-IR	Yes		****	<0.0001	9, 14
WT vs. S6k-IR	Yes		ns	0.9261	9, 14
WT vs. Akt-IR	Yes		ns	0.7088	9, 10
WT vs. Akt-OE	Yes		*	0.0126	9, 11
WT vs. S6k-OE	Yes		****	<0.0001	9,10
WT vs. Cul1-IR	Yes		ns	0.9965	9,12
**Fig 2A (P-S6k IHC)**					
WT vs. S6k-IR	No	Mann-Whitney test	****	<0.0001	14, 12
WT vs. CCT5-IR	Yes	One-way ANOVA & Tukey's	***	0.0008	9, 9
WT vs. Raptor-IR	Yes	One-way ANOVA & Dunnett's	**	0.0099	14, 9
WT vs. Raptor-OE	Yes	One-way ANOVA & Dunnett's	ns	0.9612	14, 16
WT vs. Cullin1-IR	Yes	Unpaired t-test	*	0.0454	14, 13

**Fig 2B (Raptor IHC)**		Kruskal-Wallis & Dunn's			
Raptor-OE vs. CCT5-IR	Yes		****	<0.0001	17, 13
Raptor-OE vs. Raptor-OE;CCT5-IR	Yes		***	0.0002	17, 10
CCT5-IR vs. Raptor-OE;CCT5-IR	No		ns	>0.9999	15, 10
WT vs. CCT5-IR	Yes		*	0.0355	15, 13
WT vs. Raptor-OE;CCT5-IR	Yes		ns	0.2341	15, 10
**Fig 2C (P-S6k IHC)**		Kruskal-Wallis & Dunn's			
WT vs. CCT5-IR	No		****	<0.0001	13, 10
WT vs. S6k-OE;CCT5-IR	Yes		****	<0.0001	13, 11
CCT5-IR vs. S6k-OE;CCT5-IR	No		ns	0.9773	10, 11
**Fig 2E (TDL)**		One-way ANOVA & Šídák's			
WT vs. CCT3-IR;Cul1-IR	Yes		**	0.0020	13, 10
WT vs. S6k-IR;Cul1-IR	Yes		ns	0.9864	13, 10
WT vs S6k-OE;CCT5-IR	Yes		****	<0.0001	13, 10
WT vs. S6k-OE;Cul1-IR	Yes		****	0.9864	13, 7
CCT3-IR vs. CCT3-IR;Cul1-IR	Yes		ns	0.9979	10, 10
CCT3-IR;Cul1-IR vs Cul1-IR	Yes		****	<0.0001	10, 13
Cul1-IR vs S6k-IR;Cul1-IR	Yes		ns	0.1209	10, 10
S6k-IR;Cul1-IR vs S6k-IR	Yes		****	<0.0001	10, 13
CCT5-IR vs. S6k-OE;CCT5-IR	Yes		ns	0.0589	10, 12
S6k-OE;CCT5-IR vs S6k-OE	Yes		****	<0.0001	10, 10
S6k-OE vs. S6k-OE;Cul1-IR	Yes		ns	0.4671	7, 10
Cul1-IR vs. S6k-OE;Cul1-IR	Yes		ns	0.9471	13, 7
**Fig 2F (Sholl Maximum)**		One-way ANOVA & Šídák's			
WT vs. S6k-IR;Cul1-IR	Yes		ns	0.4357	9, 10
S6k-IR vs. S6k-IR;Cul1-IR	Yes		**	0.0011	14, 10
S6k-IR;Cul1-IR vs. Cul1-IR	Yes		*	0.0119	10, 12
**Fig 3A (IHC acet tub)**					
WT vs. CCT3-IR	Yes	One-way ANOVA & Dunnett's	****	<0.0001	14, 11
WT vs. CCT5-IR	Yes	One-way ANOVA & Dunnett's	**	<0.0001	14, 13
WT vs. Raptor-IR	No	One-way ANOVA & Dunnett's	***	0.0006	14, 9
WT vs. S6k-IR	No	Mann-Whitney	***	0.0008	14, 12

WT vs. Akt-IR	Yes	One-way ANOVA & Dunnett's	*	0.0148	16, 14
WT vs. Cullin1-IR	Yes	Unpaired t-test	ns	0.2324	14, 13
WT vs. S6k-OE	Yes	One-way ANOVA & Dunnett's	ns	0.9584	10, 9
WT vs. Akt-OE	Yes	One-way ANOVA & Dunnett's	ns	0.6653	16, 12
**Fig 3A (IHC Futsch)**					
WT vs. CCT3-IR	Yes	Unpaired t-test	***	0.0002	13, 12
WT vs. CCT5-IR	Yes	Unpaired t-test	*	0.0398	9, 5
WT vs. Raptor-IR	Yes	Unpaired t-test	**	0.0089	14, 11
WT vs. S6k-IR	Yes	Unpaired t-test	*	0.0427	13, 13
WT vs. Akt-IR	Yes	One-way ANOVA & Dunnett's	***	0.0002	14, 13
WT vs. Cullin1-IR	Yes	Unpaired t-test	ns	0.0767	10, 13
WT vs. S6k-OE	Yes	One-way ANOVA & Dunnett's	***	0.0004	16, 15
WT vs. Akt-OE	Yes	Unpaired t-test	**	0.0013	10, 13
**Fig 3C (*mCherry::Jupiter*)**		Two-Way ANOVA & Tukey's			
20 µm: WT vs. CCT3-IR			****	<0.0001	12, 10
20 µm: WT vs. CCT5-IR			****	<0.0001	12, 10
20 µm: WT vs. Raptor-IR			****	<0.0001	12, 10
20 µm: WT vs. S6k-IR			**	0.0035	12, 11
20 µm: WT vs. Akt-IR			**	0.0040	12, 10
20 µm: WT vs. Cullin1-IR			****	<0.0001	12, 11
20 µm: WT vs. S6k-OE			ns	0.8037	12, 10
20 µm: WT vs. Akt-OE			ns	>0.9999	12, 10
40 µm: WT vs. CCT3-IR			****	<0.0001	12, 10
40 µm: WT vs. CCT5-IR			****	<0.0001	12, 10
40 µm: WT vs. Raptor-IR			****	<0.0001	12, 10
40 µm: WT vs. S6k-IR			**	0.0035	12, 11
40 µm: WT vs. Akt-IR			**	0.0040	12, 10
40 µm: WT vs. Cullin1-IR			****	<0.0001	12, 11
40 µm: WT vs. S6k-OE			ns	0.8037	12, 10
40 µm: WT vs. Akt-OE			ns	>0.9999	12, 10
60 µm: WT vs. CCT3-IR			****	<0.0001	12, 10
60 µm: WT vs. CCT5-IR			****	<0.0001	12, 10
60 µm: WT vs. Raptor-IR			****	<0.0001	12, 10
60 µm: WT vs. S6k-IR			**	0.0035	12, 11
60 µm: WT vs. Akt-IR			**	0.0040	12, 10
60 µm: WT vs. Cullin1-IR			****	<0.0001	12, 11
60 µm: WT vs. S6k-OE			ns	0.8037	12, 10

60 µm: WT vs. Akt-OE			ns	>0.9999	12, 10
80 µm: WT vs. CCT3-IR			****	<0.0001	12, 10
80 µm: WT vs. CCT5-IR			****	<0.0001	12, 10
80 µm: WT vs. Raptor-IR			****	<0.0001	12, 10
80 µm: WT vs. S6k-IR			**	0.0035	12, 11
80 µm: WT vs. Akt-IR			**	0.0040	12, 10
80 µm: WT vs. Cullin1-IR			****	<0.0001	12, 11
80 µm: WT vs. S6k-OE			ns	0.8037	12, 10
80 µm: WT vs. Akt-OE			ns	>0.9999	12, 10
100 µm: WT vs. CCT3-IR			****	<0.0001	12, 10
100 µm: WT vs.CCT5-IR			****	<0.0001	12, 10
100 µm: WT vs. Raptor-IR			****	<0.0001	12, 10
100 µm: WT vs. S6k-IR			**	0.0035	12, 11
100 µm: WT vs. Akt-IR			**	0.0040	12, 10
100 µm: WT vs. Cullin1-IR			****	<0.0001	12, 11
100 µm: WT vs. S6k-OE			ns	0.8037	12, 10
100 µm: WT vs. Akt-OE			ns	>0.9999	12, 10
120 µm: WT vs. CCT3-IR			****	<0.0001	12, 10
120 µm: WT vs. CCT5-IR			****	<0.0001	12, 10
120 µm: WT vs. Raptor-IR			****	<0.0001	12, 10
120 µm: WT vs. S6k-IR			**	0.0035	12, 11
120 µm: WT vs. Akt-IR			**	0.0040	12, 10
120 µm: WT vs. Cullin1-IR			****	<0.0001	12, 11
120 µm: WT vs. S6k-OE			ns	0.8037	12, 10
120 µm: WT vs. Akt-OE			ns	>0.9999	12, 10
140 µm: WT vs. CCT3-IR			****	<0.0001	12, 10
140 µm: WT vs. CCT5-IR			****	<0.0001	12, 10
140 µm: WT vs. Raptor-IR			****	<0.0001	12, 10
140 µm: WT vs. S6k-IR			**	0.0035	12, 11
140 µm: WT vs. Akt-IR			**	0.0040	12, 10
140 µm: WT vs. Cullin1-IR			****	<0.0001	12, 11
140 µm: WT vs. S6k-OE			ns	0.8037	12, 10
140 µm: WT vs. Akt-OE			ns	>0.9999	12, 10
160 µm: WT vs. CCT3-IR			****	<0.0001	12, 10
160 µm: WT vs. CCT5-IR			****	<0.0001	12, 10
160 µm: WT vs. Raptor-IR			****	<0.0001	12, 10
160 µm: WT vs. S6k-IR			**	0.0035	12, 11
160 µm: WT vs. Akt-IR			**	0.0040	12, 10
160 µm: WT vs. Cullin1-IR			****	<0.0001	12, 11
160 µm: WT vs. S6k-OE			ns	0.8037	12, 10
160 µm: WT vs. Akt-OE			ns	>0.9999	12, 10
180 µm: WT vs. CCT3-IR			****	<0.0001	12, 10
180 µm: WT vs. CCT5-IR			****	<0.0001	12, 10
180 µm: WT vs. Raptor-IR			****	<0.0001	12, 10
180 µm: WT vs. S6k-IR			**	0.0035	12, 11
180 µm: WT vs. Akt-IR			**	0.0040	12, 10

180 µm: WT vs. Cullin1-IR			****	<0.0001	12, 11
180 µm: WT vs. S6k-OE			ns	0.8037	12, 10
180 µm: WT vs. Akt-OE			ns	>0.9999	12, 10
200 µm: WT vs. CCT3-IR			****	<0.0001	12, 10
200 µm: WT vs. CCT5-IR			****	<0.0001	12, 10
200 µm: WT vs. Raptor-IR			****	<0.0001	12, 10
200 µm: WT vs. S6k-IR			**	0.0035	12, 11
200 µm: WT vs. Akt-IR			**	0.0040	12, 10
200 µm: WT vs. Cullin1-IR			****	<0.0001	12, 11
200 µm: WT vs. S6k-OE			ns	0.8037	12, 10
200 µm: WT vs. Akt-OE			ns	>0.9999	12, 10
220 µm: WT vs. CCT3-IR			****	<0.0001	12, 10
220 µm: WT vs. CCT5-IR			****	<0.0001	12, 10
220 µm: WT vs. Raptor-IR			****	<0.0001	12, 10
220 µm: WT vs. S6k-IR			**	0.0035	12, 11
220 µm: WT vs. Akt-IR			**	0.0040	12, 10
220 µm: WT vs. Cullin1-IR			****	<0.0001	12, 11
220 µm: WT vs. S6k-OE			ns	0.8037	12, 10
220 µm: WT vs. Akt-OE			ns	>0.9999	12, 10
240 µm: WT vs. CCT3-IR			****	<0.0001	12, 10
240 µm: WT vs. CCT5-IR			****	<0.0001	12, 10
240 µm: WT vs. Raptor-IR			****	<0.0001	12, 10
240 µm: WT vs. S6k-IR			**	0.0035	12, 11
240 µm: WT vs. Akt-IR			**	0.0040	12, 10
240 µm: WT vs. Cullin1-IR			****	<0.0001	12, 11
240 µm: WT vs. S6k-OE			ns	0.8037	12, 10
240 µm: WT vs. Akt-OE			ns	>0.9999	12, 10
260 µm: WT vs. CCT3-IR			****	<0.0001	12, 10
260 µm: WT vs. CCT5-IR			****	<0.0001	12, 10
260 µm: WT vs. Raptor-IR			****	<0.0001	12, 10
260 µm: WT vs. S6k-IR			**	0.0035	12, 11
260 µm: WT vs. Akt-IR			**	0.0040	12, 10
260 µm: WT vs. Cullin1-IR			****	<0.0001	12, 11
260 µm: WT vs. S6k-OE			ns	0.8037	12, 10
260 µm: WT vs. Akt-OE			ns	>0.9999	12, 10
280 µm: WT vs. CCT3-IR			****	<0.0001	12, 10
280 µm: WT vs. CCT5-IR			****	<0.0001	12, 10
280 µm: WT vs. Raptor-IR			****	<0.0001	12, 10
280 µm: WT vs. S6k-IR			**	0.0035	12, 11
280 µm: WT vs. Akt-IR			**	0.0040	12, 10
280 µm: WT vs. Cullin1-IR			****	<0.0001	12, 11
280 µm: WT vs. S6k-OE			ns	0.8037	12, 10
280 µm: WT vs. Akt-OE			ns	>0.9999	12, 10
300 µm: WT vs. CCT3-IR			****	<0.0001	12, 10
300 µm: WT vs. CCT5-IR			****	<0.0001	12, 10
300 µm: WT vs. Raptor-IR			****	<0.0001	12, 10

300 µm: WT vs. S6k-IR			**	0.0035	12, 11
300 µm: WT vs. Akt-IR			**	0.0040	12, 10
300 µm: WT vs. Cullin1-IR			****	<0.0001	12, 11
300 µm: WT vs. S6k-OE			ns	0.8037	12, 10
300 µm: WT vs. Akt-OE			ns	>0.9999	12, 10
320 µm: WT vs. CCT3-IR			****	<0.0001	12, 10
320 µm: WT vs. CCT5-IR			****	<0.0001	12, 10
320 µm: WT vs. Raptor-IR			****	<0.0001	12, 10
320 µm: WT vs. S6k-IR			**	0.0035	12, 11
320 µm: WT vs. Akt-IR			**	0.0040	12, 10
320 µm: WT vs. Cullin1-IR			****	<0.0001	12, 11
320 µm: WT vs. S6k-OE			ns	0.8037	12, 10
320 µm: WT vs. Akt-OE			ns	>0.9999	12, 10
340 µm: WT vs. CCT3-IR			****	<0.0001	12, 10
340 µm: WT vs. CCT5-IR			****	<0.0001	12, 10
340 µm: WT vs. Raptor-IR			****	<0.0001	12, 10
340 µm: WT vs. S6k-IR			**	0.0035	12, 11
340 µm: WT vs. Akt-IR			**	0.0040	12, 10
340 µm: WT vs. Cullin1-IR			****	<0.0001	12, 11
340 µm: WT vs. S6k-OE			ns	0.8037	12, 10
340 µm: WT vs. Akt-OE			ns	>0.9999	12, 10
360 µm: WT vs. CCT3-IR			****	<0.0001	12, 10
360 µm: WT vs. CCT5-IR			****	<0.0001	12, 10
360 µm: WT vs. Raptor-IR			****	<0.0001	12, 10
360 µm: WT vs. S6k-IR			**	0.0035	12, 11
360 µm: WT vs. Akt-IR			**	0.0040	12, 10
360 µm: WT vs. Cullin1-IR			****	<0.0001	12, 11
360 µm: WT vs. S6k-OE			ns	0.8037	12, 10
360 µm: WT vs. Akt-OE			ns	>0.9999	12, 10
380 µm: WT vs. CCT3-IR			****	<0.0001	12, 10
380 µm: WT vs. CCT5-IR			****	<0.0001	12, 10
380 µm: WT vs. Raptor-IR			****	<0.0001	12, 10
380 µm: WT vs. S6k-IR			**	0.0035	12, 11
380 µm: WT vs. Akt-IR			**	0.0040	12, 10
380 µm: WT vs. Cullin1-IR			****	<0.0001	12, 11
380 µm: WT vs. S6k-OE			ns	0.8037	12, 10
380 µm: WT vs. Akt-OE			ns	>0.9999	12, 10
400 µm: WT vs. CCT3-IR			****	<0.0001	12, 10
400 µm: WT vs. CCT5-IR			****	<0.0001	12, 10
400 µm: WT vs. Raptor-IR			****	<0.0001	12, 10
400 µm: WT vs. S6k-IR			**	0.0035	12, 11
400 µm: WT vs. Akt-IR			**	0.0040	12, 10
400 µm: WT vs. Cullin1-IR			****	<0.0001	12, 11
400 µm: WT vs. S6k-OE			ns	0.8037	12, 10
400 µm: WT vs. Akt-OE			ns	>0.9999	12, 10
420 µm: WT vs. CCT3-IR			****	<0.0001	12, 10

420 µm: WT vs. CCT5-IR			****	<0.0001	12, 10
420 µm: WT vs. Raptor-IR			****	<0.0001	12, 10
420 µm: WT vs. S6k-IR			**	0.0035	12, 11
420 µm: WT vs. Akt-IR			**	0.0040	12, 10
420 µm: WT vs. Cullin1-IR			****	<0.0001	12, 11
420 µm: WT vs. S6k-OE			ns	0.8037	12, 10
420 µm: WT vs. Akt-OE			ns	>0.9999	12, 10
440 µm: WT vs. CCT3-IR			****	<0.0001	12, 10
440 µm: WT vs. CCT5-IR			****	<0.0001	12, 10
440 µm: WT vs. Raptor-IR			****	<0.0001	12, 10
440 µm: WT vs. S6k-IR			**	0.0035	12, 11
440 µm: WT vs. Akt-IR			**	0.0040	12, 10
440 µm: WT vs. Cullin1-IR			****	<0.0001	12, 11
440 µm: WT vs. S6k-OE			ns	0.8037	12, 10
440 µm: WT vs. Akt-OE			ns	>0.9999	12, 10
460 µm: WT vs. CCT3-IR			****	<0.0001	12, 10
460 µm: WT vs. Raptor-IR			****	<0.0001	12, 10
460 µm: WT vs. Akt-IR			**	0.0040	12, 10
460 µm: WT vs. Cullin1-IR			****	<0.0001	12, 11
460 µm: WT vs. S6k-OE			ns	0.8037	12, 11
460 µm: WT vs. Akt-OE			ns	>0.9999	12, 10
480 µm: WT vs. Akt-IR			**	0.0040	12, 10
480 µm: WT vs. Cullin1-IR			****	<0.0001	12, 11
480 µm: WT vs. S6k-OE			ns	0.8037	12, 11
480 µm: WT vs. Akt-OE			ns	>0.9999	12, 10
500 µm: WT vs. Akt-IR			**	0.0040	12, 10
500 µm: WT vs. Cullin1-IR			****	<0.0001	12, 11
500 µm: WT vs. S6k-OE			ns	0.8037	12, 11
500 µm: WT vs. Akt-OE			ns	>0.9999	12, 10
**Fig 4D (HTT *mCherry::Jupiter*)**		Two-Way ANOVA & Tukey's			
40 µm: HTT20 vs. HTT50			ns	0.9996	10,9
40 µm: HTT20 vs. HTT93			****	<0.0001	10,11
80 µm: HTT20 vs. HTT50			ns	0.9996	10,9
80 µm: HTT20 vs. HTT93			****	<0.0001	10,11
120 µm: HTT20 vs. HTT50			ns	0.9996	10,9
120 µm: HTT20 vs. HTT93			****	<0.0001	10,11
160 µm: HTT20 vs. HTT50			ns	0.9996	10,9
160 µm: HTT20 vs. HTT93			****	<0.0001	10,11
200 µm: HTT20 vs. HTT50			ns	0.9996	10,9
200 µm: HTT20 vs. HTT93			****	<0.0001	10,11
240 µm: HTT20 vs. HTT50			ns	0.9996	10,9
240 µm: HTT20 vs. HTT93			****	<0.0001	10,11
280 µm: HTT20 vs. HTT50			ns	0.9996	10,9
280 µm: HTT20 vs. HTT93			****	<0.0001	10,11

320 µm: HTT20 vs. HTT50			ns	0.9996	10,9
320 µm: HTT20 vs. HTT93			****	<0.0001	10,11
360 µm: HTT20 vs. HTT50			ns	0.9996	10,9
360 µm: HTT20 vs. HTT93			****	<0.0001	10,11
400 µm: HTT20 vs. HTT50			ns	0.9996	10,9
400 µm: HTT20 vs. HTT93			****	<0.0001	10,11
440 µm: HTT20 vs. HTT50			ns	0.9996	10,9
440 µm: HTT20 vs. HTT93			****	<0.0001	10,11
**Fig 4E (HTT TDL)**		One-way ANOVA & Šídák’s			
WT vs. HTTQ25	Yes		*	0.0103	11, 11
WT vs. HTT96	Yes		*	0.0450	11, 16
WT vs. HTTQ96;CCT5-IR	Yes		****	<0.0001	11, 16
WT vs. HTTQ96;Cul1-IR	Yes		ns	0.9254	11, 9
WT vs. HTT25;CCT5-IR	Yes		****	<0.0001	11, 9
WT vs. HTTQ25;Cul1-IR	Yes		ns	0.0764	11, 9
HTTQ25 vs. HTTQ25;CCT5-IR	Yes		****	<0.0001	11, 9
HTTQ25 vs. HTTQ25;Cul1-IR	Yes		ns	>0.9999	11, 9
HTTQ96 vs. HTTQ96;CCT5-IR	Yes		**	0.0012	16, 16
HTTQ96 vs. HTTQ96;Cul1-IR	Yes		***	0.0005	16, 9
HTTQ96;CCT5-IR vs. HTTQ25;CCT5-IR	Yes		ns	0.9999	16, 9
HTTQ96;Cul1-IR vs. HTTQ25;Cul1-IR	Yes		ns	0.9408	9, 9
CCT5-IR vs. HTT25;CCT5-IR	Yes		ns	0.4088	12, 9
CCT5-IR vs HTT96;CCT5-IR	Yes		ns	0.7603	12, 16
**Fig 4G (# mHTT IB)**		Kruskal-Wallis & Dunn’s			
HTT96 vs. HTT96;CCT5-IR	No		ns	>0.9999	15, 14
HTT96 vs. HTT96;Cul1-IR	No		ns	0.7909	15, 14
**Fig 4H (area mHTT IB)**		Kruskal-Wallis & Dunn’s			
HTT96 vs. HTT96;CCT5-IR	No		ns	0.1699	16, 16
HTT96 vs. HTT96;Cul1-IR	No		ns	>0.9999	16, 15
**Fig S1A (CCT TDL)**		One-way ANOVA & Dunnett’s			
WT vs. CCT1-IR	Yes		****	<0.0001	13, 10
WT vs. CCT2-IR	Yes		ns	0.1586	13, 10
WT vs. CCT4-IR	Yes		ns	0.6527	13, 10
WT vs. CCT6-IR	Yes		****	<0.0001	13, 10
WT vs. CCT7-IR	Yes		*	0.0388	13, 10
WT vs. CCT8-IR	Yes		****	<0.0001	13, 11

**Fig S1B (CCT4 TDL)**					
40A FRT vs. CCT4^{KG09280}	No	Mann-Whitney Test	***	0.0003	11, 8
**Fig S1D (CCT Sholl Max)**		One-way ANOVA & Dunnett's			
WT vs. CCT1-IR	Yes		**	0.0025	9, 10
WT vs. CCT2-IR	Yes		ns	0.9665	9, 10
WT vs. CCT4-IR	Yes		ns	0.5974	9, 11
WT vs. CCT6-IR	Yes		*	0.0153	9, 10
WT vs. CCT7-IR	Yes		ns	0.3998	9, 10
WT vs. CCT8-IR	Yes		*	0.0471	9, 11
**Fig S1E (CCT Sholl Radius)**		One-way ANOVA & Dunnett's			
WT vs. CCT1-IR	Yes		***	0.0001	9, 10
WT vs. CCT2-IR	Yes		ns	0.2714	9, 10
WT vs. CCT4-IR	Yes		ns	0.3769	9, 11
WT vs. CCT6-IR	Yes		***	0.0007	9, 10
WT vs. CCT7-IR	Yes		ns	>0.9999	9, 10
WT vs. CCT8-IR	Yes		ns	0.1483	9, 11
**Fig S1F (CCT5 IHC)**		One-way ANOVA & Šídák's			
WT vs. CCT4-IR	Yes		****	<0.0001	29, 23
WT vs. CCT5-IR	Yes		***	0.0002	29, 16
WT vs. CCT4-IR;CCT5-IR	Yes		****	<0.0001	29, 18
CCT4-IR vs. CCT4-IR;CCT5-IR	Yes		**	0.0021	23, 18
CCT5-IR vs. CCT4-IR;CCT5-IR	Yes		***	0.0004	16, 18
**Fig S1H (AEL TDL)**		One-way ANOVA & Šídák's			
24 hr: mean of genetic controls vs. CCT3-IR	Yes		ns	0.4890	17, 15
24 hr: mean of genetic controls vs. CCT5-IR	Yes		ns	0.4424	12, 13
48 hr: mean of genetic controls vs. CCT3-IR	Yes		ns	0.1891	18, 12
48 hr: mean of genetic controls vs. CCT5-IR	Yes		ns	0.6169	10, 12
72 hr: mean of genetic controls vs. CCT3-IR	Yes		***	0.0004	11, 14
72 hr: mean of genetic controls vs. CCT5-IR	Yes		*	0.0104	10, 12
96 hr: mean of genetic controls vs. CCT3-IR	Yes		****	<0.0001	10, 10

96 hr: mean of genetic controls vs. CCT5-IR	Yes		****	<0.0001	12, 12
72 hr CCT3-IR vs. 96 hr CCT3-IR	Yes		ns	0.6523	14, 10
72 hr CCT5-IR vs. 96 hr CCT5-IR	Yes		ns	0.5468	12, 12
**Fig S1I (CCT OE TDL)**		One-way ANOVA & Šídák's			
WT vs. CCT2-OE	Yes		ns	0.9917	13, 10
WT vs. drosCCT4	Yes		ns	0.6067	13, 10
dcas9 vs. CCT5 TOE	Yes		ns	0.2882	13, 11
**Fig S2A (Raptor fluorescence IHC)**					
WT vs. CCT5-IR	Yes	One-way ANOVA & Dunnett's	****	<0.0001	11, 14
WT vs. Raptor-IR	Yes	One-way ANOVA & Dunnett's	****	<0.0001	16, 16
WT vs. Raptor-OE	Yes	One-way ANOVA & Dunnett's	***	0.0005	16, 14
**Fig S2C (S6k IHC)**		Unpaired t-test			
WT vs. S6k-IR	Yes		**	0.0071	9, 8
**Fig S2E (P-Akt IHC)**		Unpaired t-test			
WT vs. Akt-IR	Yes		****	<0.0001	11, 12
**Fig S2G (Cullin1 IHC)**		Unpaired t-test			
WT vs. Cullin1-IR	Yes		**	0.0013	15, 14
**Fig S3C (BtubIIA fluorescence)**					
WT vs. CCT3-IR	Yes	One-way ANOVA & Dunnett's	****	<0.0001	14, 11
WT vs. CCT5-IR	Yes	One-way ANOVA & Dunnett's	****	<0.0001	14, 15
WT vs. S6k-IR	Yes	Unpaired t-test	*	0.0339	13, 13
WT vs. Raptor-IR	Yes	Unpaired t-test	**	0.0078	14, 11
WT vs. Akt-IR	Yes	One-way ANOVA & Dunnett's	**	0.0055	16, 14
WT vs. Akt-OE	Yes	One-way ANOVA & Dunnett's	ns	0.4915	16, 12
WT vs. Cullin1-IR	Yes	Unpaired t-test	*	0.0105	10, 13
**Fig S4B (HTT TDL)**		One-way ANOVA & Šídák's			
WT vs. HTTQ20	Yes		ns	>0.9999	11, 15
WT vs. HTTQ50	Yes		ns	0.8793	11, 13

WT vs. HTTQ93	Yes		****	<0.0001	11, 18
WT vs. HTTQ120	Yes		****	<0.0001	11, 15
**Fig S4D (HTT Futsch)**		One-way ANOVA & Tukey's			
WT vs. HTT96	Yes		****	<0.0001	11, 11
WT vs. HTT96;CCT5-IR	Yes		****	<0.0001	11, 9
HTT96 vs. HTT96;CCT5-IR	Yes		ns	>0.9999	11, 9
**Fig S4F (WT HTT)**					
WT vs. CCT5-IR	Yes	Unpaired t-test	***	0.0002	11, 10
**Fig S4G (WT HTT IHC)**		One-way ANOVA & Tukey's			
WT vs. HTT25-Cer	Yes		*	0.0117	11, 11
WT vs. HTT96-Cer	Yes		****	<0.0001	11, 11
