# Supplementary figures and images for: CCT and Cullin1 Regulate the TORC1 Pathway to Promote Dendritic Arborization in Health and Disease
Source: Cells. 2024 Jun 13;13(12):1029. doi: 10.3390/cells13121029 (PMC11201622; doi:10.3390/cells13121029)

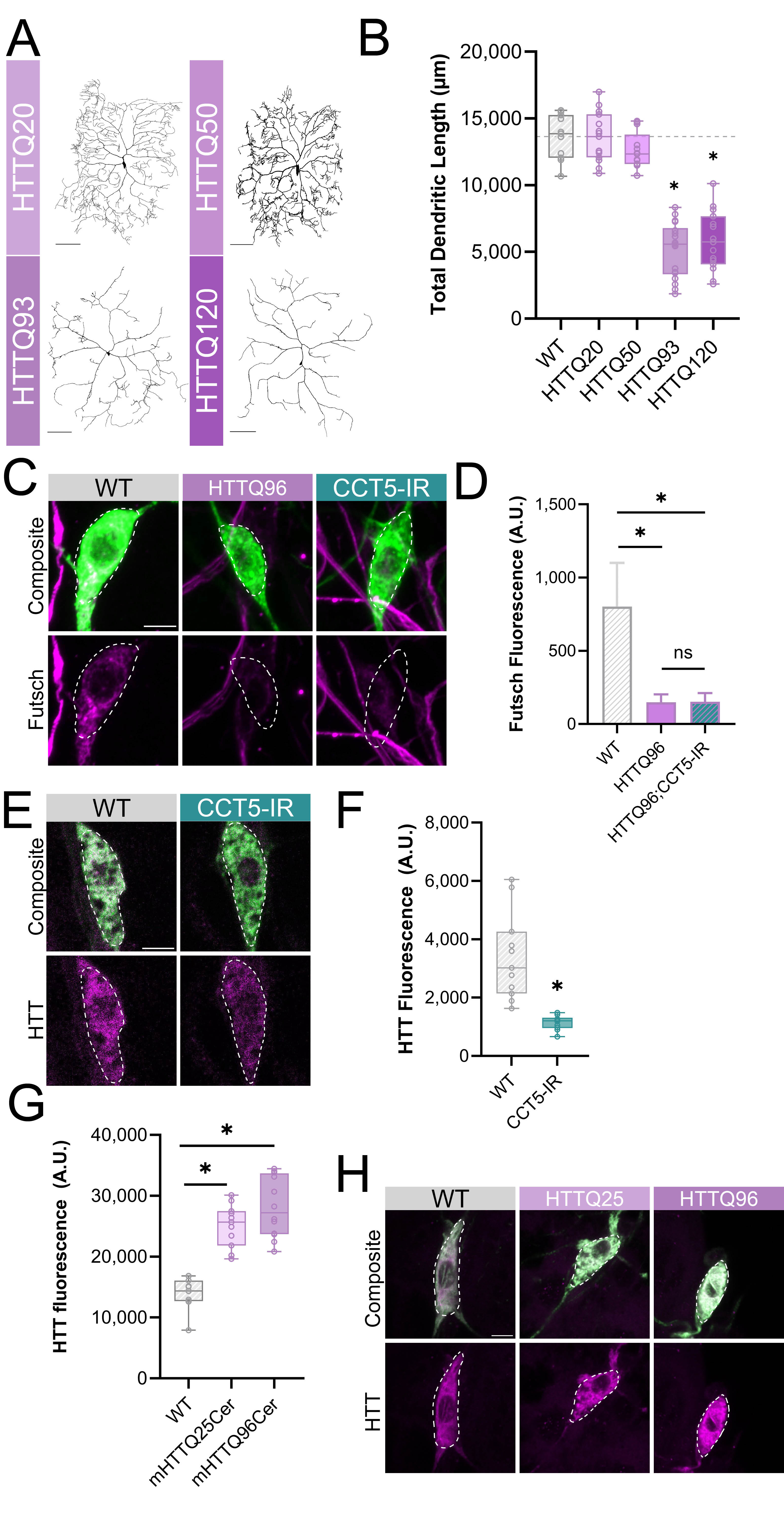

Supplement: Supplementary file 1 [file cells-13-01029-s001.zip › FigS4.tif]
